# Effect of dynamic cerebral autoregulation on the association between deep medullary vein changes and cerebral small vessel disease

**DOI:** 10.3389/fphys.2023.1037871

**Published:** 2023-04-04

**Authors:** Ling He, Zhen-Ni Guo, Yang Qu, Run-Ting Wang, Peng Zhang, Yi Yang, Hang Jin

**Affiliations:** ^1^ Stroke Center and Clinical Trial and Research Center for Stroke, Department of Neurology, The First Hospital of Jilin University, Changchun, China; ^2^ China National Comprehensive Stroke Center, Changchun, China; ^3^ Jilin Provincial Key Laboratory of Cerebrovascular Disease, Changchun, China

**Keywords:** cerebral small vessel disease, deep medullary vein, dynamic cerebral autoregulation, total cerebral small vessel disease burden, transfer function analysis, interaction effect

## Abstract

Changes in the deep medullary vein (DMV) are reported to be associated with cerebral small vessel disease (CSVD). While the mechanisms of this association are unclear, dynamic cerebral autoregulation (dCA) has been speculated to participate in this association. Thus, we aimed to verify the association between DMV changes and total CSVD burden and further investigate the effect of dCA function on this correlation. In this prospective study, 95 Asian patients aged ≥18 years were included in the final assessment. DMV scores and total CSVD burden were determined using magnetic resonance imaging sequences. Transfer function analysis was performed to analyze dCA function. Generalized linear regressions were used to assess the relationship between DMV changes and total CSVD burden as well as between DMV changes and dCA function. An interaction model was utilized to assess the effect of dCA function on the association between DMV changes and total CSVD burden. Generalized linear models showed a significant positive association between DMV changes and total CSVD burden (*p* = 0.039) and a significant negative association between DMV changes and dCA function (*p* = 0.018). The interaction model demonstrated a significant positive interaction of dCA impairment on the association between DMV changes and the total CSVD burden (*p* = 0.02). Thus, we came to the conclusion that changes in DMV were correlated independently with both CSVD and dCA impairment and furthermore, impaired dCA function play an interaction effect on the association between DMV changes and the total CSVD burden. Our results can help improve the understanding of the complex pathogenesis and progression of CSVD, thereby facilitating early intervention and treatment development.

## 1 Introduction

Cerebral small vessel disease (CSVD) is a syndrome characterized by clinical, cognitive, neuroimaging, and neuropathological findings that are thought to arise from diseases affecting the perforating cerebral arterioles, capillaries, and venules (Wardlaw et al., 2013a). CSVD is very common among the older individuals and contributes substantially to cognitive decline, dementia, depression, gait abnormalities, and bowel and bladder disturbances. The detection rate and public health burden of CSVD have increased considerably in the past few years; however, the underlying pathogenesis is poorly understood (Wardlaw et al., 2019).

The deep medullary vein (DMV) is the main part of the deep draining veins of the parenchyma. Located in the periventricular white matter, the DMV drains venous blood from the white matter towards the subependymal veins of the lateral ventricles (Kuijf et al., 2016). Advances in neuroimaging technologies have enabled DMVs to be visualized using susceptibility-weighted imaging (SWI). Since deep medullary venous collagenosis was identified in autopsy cases by Moody in 1995 (Moody et al., 1995), several studies have focused on the venous mechanisms of CSVD and have shown that DMV changes are closely related to the presence and progression of CSVD, including lacunes (Chen et al., 2020), severe perivascular spaces (Zhang et al., 2022a), brain atrophy (Ao et al., 2021; Liu et al., 2021), microbleeds (Zhang et al., 2019), and especially white matter hyperintensities (WMHs) (Keith et al., 2017; Zhang et al., 2017). However, studies on the association between DMV changes and total CSVD burden did not show completely consistent results. Xu et al. (2020) found that DMV scores were closely correlated with total CSVD burden, but Chen et al. (2020) did not find this correlation in a larger sample size. Consequently, the association between DMV changes and total CSVD burden is disputed.

The mechanisms underlying the correlation between DMV changes and CSVD are unclear, but they are thought to involve cerebral autoregulation (Keith et al., 2017; Nan et al., 2019; Shaaban et al., 2019; Zhou et al., 2020). Dynamic cerebral autoregulation (dCA) is the intrinsic ability of the brain to maintain adequate cerebral perfusion in the presence of blood pressure changes (Claassen et al., 2016). Impairment of dCA has been associated with many clinical diseases, including stroke (Guo et al., 2014), Alzheimer’s disease (Gommer et al., 2012), and obstructive sleep apnea-hypopnea syndrome (Zhang et al., 2021). In a previous study, we found that dCA impairment was associated with the severity of CSVD neuroimaging features (Liu et al., 2022). Moody et al. (1995) and Keith et al. (2017) hypothesized that DMV changes may increase venous resistance and impair cellular clearance ability, leading to impairment of cerebral autoregulation (Nan et al., 2019), decreased cerebral blood flow, and progression to CSVD. However, this speculation has not been proven.

Therefore, in this prospective observational study, we aimed to explore 1) the association between DMV changes and total CSVD burden, 2) the correlation between DMV changes and dCA, and 3) the effect of dCA on the association between DMV changes and CSVD burden.

## 2 Materials and methods

### 2.1 Study participants

This was a prospective observational study of patients with CSVD admitted to the Department of Neurology at the First Hospital of Jilin University from October 2021 to March 2022. The inclusion criteria were: 1) Asian patients aged ≥18; 2) definitely diagnosed with CSVD by at least two neurologists whose magnetic resonance imaging (MRI) met at least one feature of CSVD including recent small subcortical infarcts, lacunes, white matter hyperintensities, perivascular spaces and microbleeds (21); 3) agreed to participate in this study, and signed informed consent. The exclusion criteria were 1) intracranial lesions other than those in CSVD (such as a tumor, head trauma, hemorrhage, multiple sclerosis, and encephalitis); 2) hereditary CSVD; 3) moderate-to-severe carotid or intracranial artery stenosis (≥50%, measured with an EMS-9PB transcranial Doppler detector [Delica, Shenzhen, China] and an iU22 ultrasound [Phillips, Andover, MA]) or occlusion; 4) patients with insufficient bilateral temporal bone window for insonation of the middle cerebral artery; 5) participants who were unable to cooperate dCA evaluation; and 6) myocardial infarction, atrial fibrillation and heart failure that could affect cerebral hemodynamics.

### 2.2 Ethics statement

This study was approved by the Ethics Committee of the First Hospital of Jilin University (22K047-001). Written informed consent was obtained from all participants before the investigation, and they had the right to withdraw from the study at any time. All clinical investigations were conducted in accordance with the principles of the Declaration of Helsinki.

### 2.3 Clinical assessment

The demographic and clinical data collected for each participant included information on age, sex, current smoking and drinking habits, and histories of hypertension, diabetes, hyperlipidemia, and coronary heart disease. Hypertension was defined as systolic blood pressure ≥140 mmHg, diastolic blood pressure ≥90 mmHg, or self-reported hypertension (Al-Makki et al., 2022). Hyperlipidemia was defined as total cholesterol level >5.2 mmol/L, low-density lipoprotein level >3.36 mmol/L, taking anti-dyslipidemia medication, or self-reported hyperlipidemia (Mach et al., 2019). Diabetes mellitus was defined as fasting blood glucose level ≥7.0 mmol/L or treatment with antidiabetic drugs or insulin (Riddle et al., 2021). Coronary heart disease was defined as self-reported coronary heart disease. In addition, the following laboratory examination results were recorded: fasting blood glucose, total cholesterol, uric acid, folic acid, and homocysteine levels.

### 2.4 MRI assessment

All participants underwent brain MRI scans using a 3.0T MR scanner (Siemens; Erlangen, Germany). Lacunes of presumed vascular origin were defined as a round or ovoid, subcortical, fluid-filled cavity with a central cerebrospinal fluid-like hypointensity and a surrounding rim of hyperintensity on fluid-attenuated inversion recovery imaging sequences (Wardlaw et al., 2013a). WMHs of presumed vascular origin were defined as being hypointense or isointense on T1-weighted imaging and hyperintense on T2-weighted imaging and fluid-attenuated inversion recovery imaging sequences (Wardlaw et al., 2013b). WMH was graded according to the modified Fazekas scale (Fazekas et al., 1987). Cerebral microbleeds were defined as small areas of signal voids on SWI (generally 2–5 mm in diameter, but up to 10 mm) (Wardlaw et al., 2013a). An enlarged perivascular space was defined as a fluid-filled space that followed the typical course of a vessel and had signal intensity similar to that of cerebrospinal fluid on all MRI sequences (Wardlaw et al., 2013b). The total CSVD burden was the sum of scores for the presence (each presence adding 1 point) of lacunes, cerebral microbleeds, perivascular spaces (moderate to severe in the basal ganglia), and WMH (severe in periventricular WMH; moderate-to-severe in deep cortical WMH) (Staals et al., 2014)). All MRI images were analyzed independently by two trained neurologists (QY and ZP) who were blinded to the clinical data. If there was any disagreement, the neurologists discussed the issue with a senior neurologist to reach the final result.

### 2.5 Measurement of DMV

**FIGURE 1:**
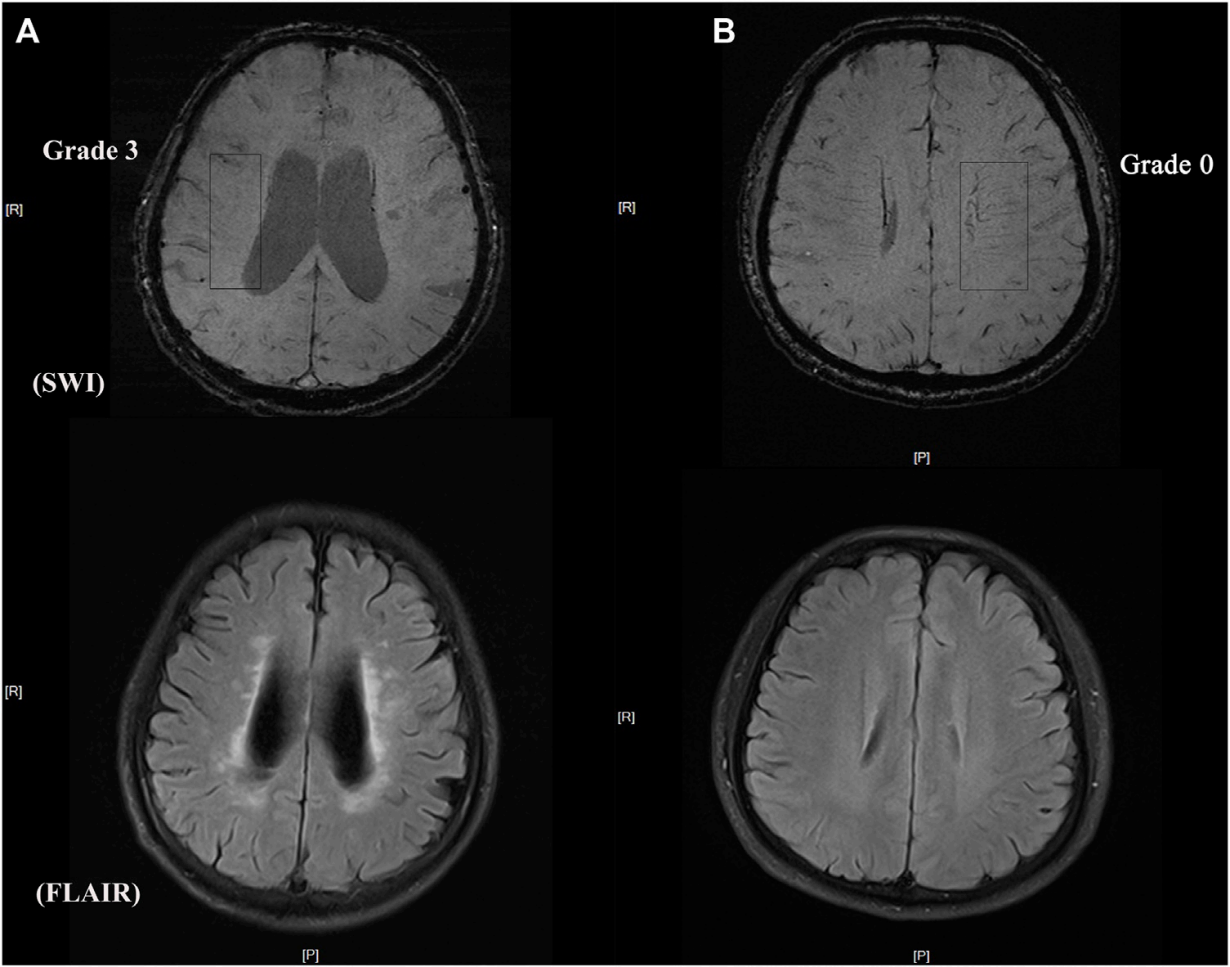
Representative images of DMV and CSVD burden **(A)** DMV score = grade 3 with heavy white matter hyperintensity burden **(B)** DMV score = grade 0 with light white matter hyperintensity burden.

The number of DMVs was assessed visually using SWI based on a brain region-based DMV visual score protocol (Zhang et al., 2017) by two trained neurologists (HL and WRT) who were blinded to all clinical data. Briefly, a region of interest from the level of the ventricles immediately above the basal ganglia to the level of the ventricles that immediately disappeared was selected in each cerebral hemisphere. Three regions (frontal, parietal, and occipital) were identified in each region of interest according to the DMV anatomy. DMVs crossing a region of interest were quantified using a four-point grading scale (Grade 0: each vein was continuous and had a homogeneous signal; Grade 1: each vein was continuous, but one or more veins had an inhomogeneous signal; Grade 2: one or more veins were not continuous and presented with spot-like hypointensity; Grade 3: no observed vein was continuous). The total number of DMVs was the sum of the grades for the three regions in each hemisphere. Representative images of DMV are shown in Figure 1.

### 2.6 Measurement of dCA

To measure dCA, we used transfer function analysis (Claassen et al., 2016), which was recommended by the Cerebral Autoregulation Research Network in 2016. To minimize measurement errors, all participants were asked to avoid alcohol, coffee, chocolate, nicotine, and physical exercise for at least 12 h before the dCA measurement and to maintain a relaxed supine position for 10 min before the dCA measurement. The measurement was performed in a special laboratory with the temperature controlled at 22°C–24°C, and visual and auditory stimuli were minimized. A physician who specializes in neurovascular ultrasound, and was blinded to all participants’ information, performed the measurement. Baseline blood pressure was measured in the brachial artery using an automatic blood pressure monitor (Omron 711). A 2 MHz transcranial Doppler capnograph (MultiDop X2, DWL; Sipplingen, Germany) was placed at the bilateral anterior temporal windows to record the cerebral blood flow velocity of the bilateral middle cerebral arteries; a servo-controlled plethysmograph (Finometer Model 1, FMS; Amsterdam, Netherlands) simultaneously recorded the continuous arterial blood pressure. To maintain physiological conditions during the measurement and obtain uninterrupted high-quality data, all participants were asked to stay awake and avoid speaking and body movements for at least 5 min.

### 2.6 Analysis of dCA data

The dCA data were processed using MATLAB (MathWorks; Natick, MA, United States). After using a cross-correlation function to remove possible time lags, beat-to-beat alignment of the data was achieved. A third-order low-pass Butterworth filter (cutoff at 0.5 Hz) was used for anti-aliasing before downsampling the data to 1 Hz. The obtained data were calculated using transfer function analysis, which was introduced in our previous research (Xiong et al., 2017). Briefly, two parameters were derived from the analysis to represent the autoregulation function after analysis: phase and gain, respectively. The phase provides a measure of the temporal difference between cerebral blood flow velocity oscillations in relation to arterial blood pressure (a lower phase indicates impaired dCA) (Claassen et al., 2016). The gain quantifies the damping effect of the CA on the magnitude of the oscillations in blood pressure (a higher gain indicates impaired dCA) (Claassen et al., 2016). These two autoregulatory parameters were obtained from three separate frequency ranges: very low frequency (0.02–0.07 Hz), low frequency (0.07–0.20 Hz), and high frequency (0.2–0.5 Hz). Based on our previous study (Liu et al., 2022), these two parameters obtained at low frequency have greater stability and are more related to CSVD. Therefore, we chose the phase and gain obtained at low frequencies to represent the function of the dCA. Another parameter, coherence, was used to evaluate the validity of the gain and phase. Only when coherence is > 0.3, can autoregulatory parameters be used in subsequent statistical analysis (Claassen et al., 2016); otherwise, they are deleted.

### 2.7 Statistical analysis

All data were analyzed using STATA version 13.0 (College Station, TX, United States). For continuous variables, a one-sample Kolmogorov–Smirnov test was performed to assess the normality of the data distribution. Data for normally distributed continuous variables are presented as mean ± standard deviation; data for non-normally distributed continuous variables are expressed as medians with interquartile ranges. Categorical variables are reported as frequency (percentage). To compare participant characteristics between groups, t-tests or Mann–Whitney U-tests were used for continuous variables, and Pearson’s chi-square test or Fisher’s exact probability test was used for categorical variables. To explore the relationship between DMV and dCA, as well as between DMV and total CSVD burden, generalized linear regression analyses were performed. Interaction analysis was performed to evaluate the effect of dCA on the association between the DMV scores and total CSVD burden. Statistical significance was set at *p* < 0.05.

## 3 Results

**FIGURE 2:**
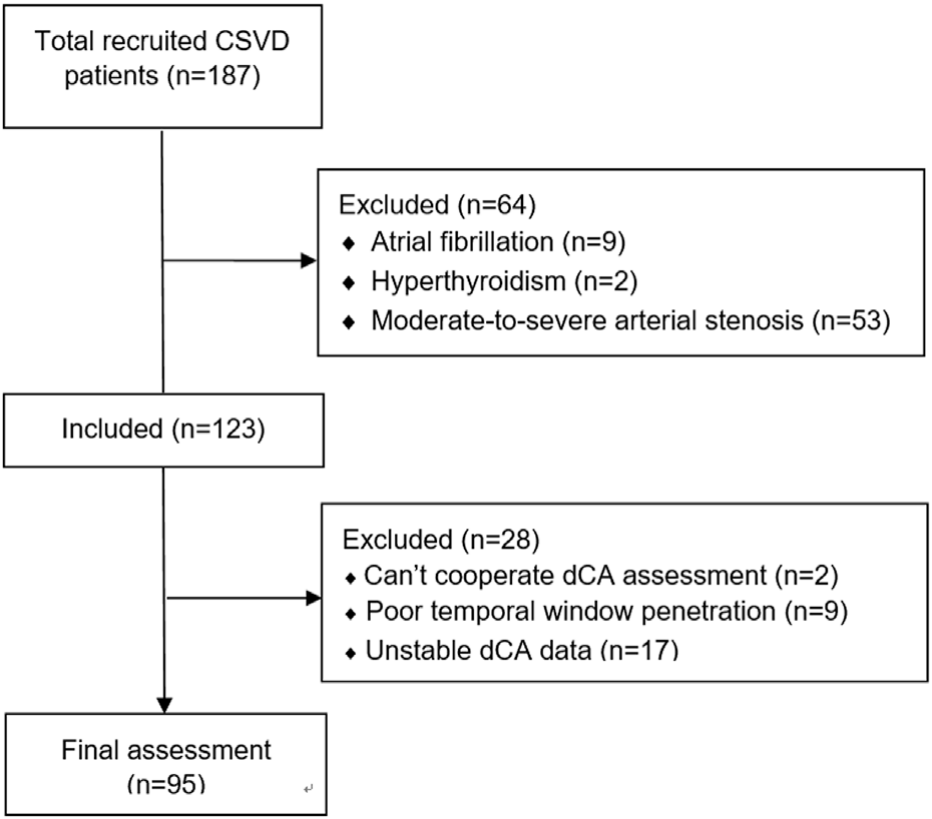
Flow chart of this study.

Overall, 187 patients with CSVD were recruited initially. After excluding arterial stenosis and disease histories that affect dCA, 123 patients were included in the study; 28 patients were excluded owing to unstable dCA data. Finally, 95 patients were included in the data analysis. A detailed flowchart is shown in Figure 2.

### 3.1 Baseline characteristics

**FIGURE 3:**
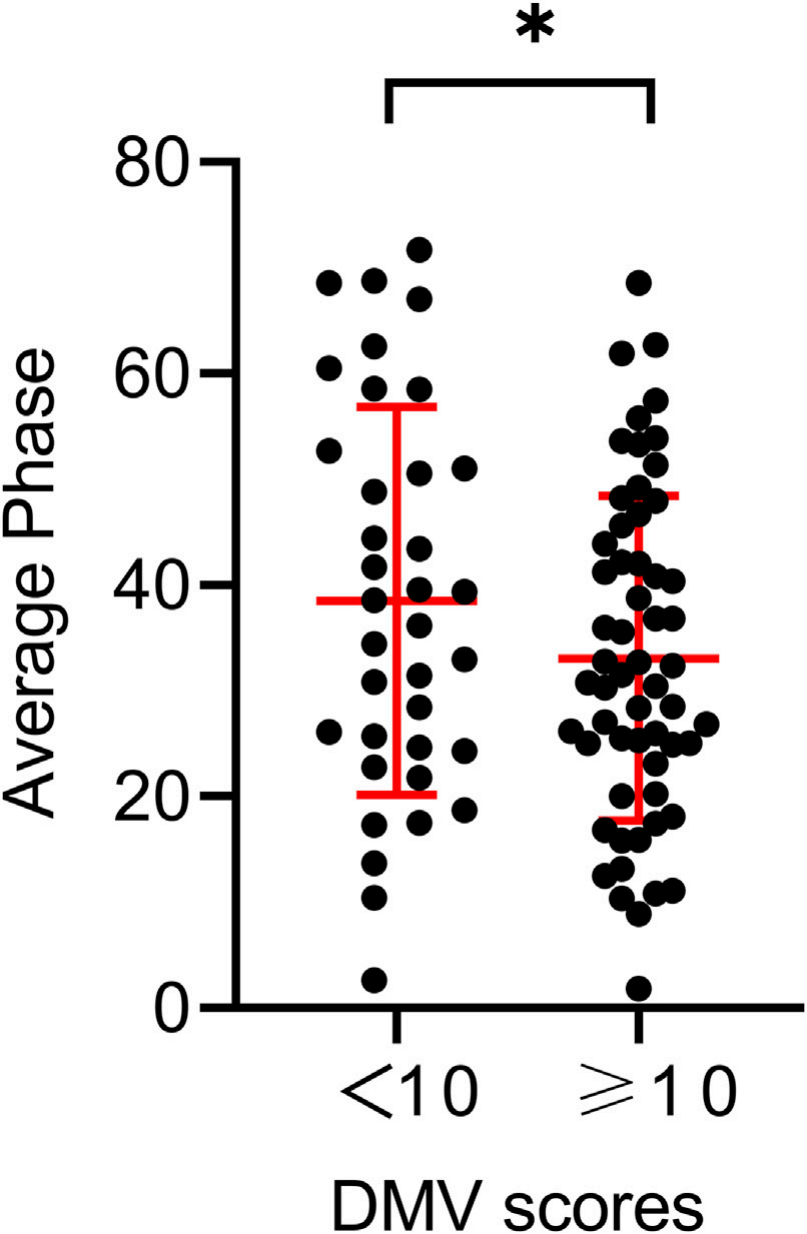
Comparison of the average phase between different DMV score groups. *p < 0.05.

Table 1 shows the baseline characteristics of the participants and the different DMV score groups. The mean age of the patients was 56.3 ± 10.6 years; 83 (87.4%) were men. Hypertension was observed in 58 (61.1%) participants, diabetes mellitus in 28 (29.5%), and hyperlipidemia in 51 (53.7%). The mean National Institutes of Health Stroke Scale score at admission was 4.6 ± 3.6. The total CSVD scores of the patients included 7 with 0 points (7.4%), 23 with 1 point (24.2%), 30 with 2 points (31.6%), 20 with 3 points (21.0%), and 15 with 4 points (15.8%). When stratifying total DMV scores across their median values in the univariate analysis, the prevalence of hypertension and the average bilateral phase showed differences among the DMV score groups (*p* = 0.022, *p* = 0.028, respectively). Individuals with total DMV scores below the mean value had a higher average bilateral phase than those with DMV scores above the mean value (Figure 3). The average bilateral gain in individuals with total DMV scores below the mean value was lower than that in those with DMV scores above the mean value, but the difference was not statistically significant (*p* = 0.141).

**TABLE 1:** Baseline characteristics of total patients and patients across categories of DMV scores.

	Total DMV scores <10 (n = 37)	Total DMV scores ≥10 (n = 58)	Total (n = 95)	P
Age (year)	55.9 ± 9.7	56.6 ± 11.2	56.3 ± 10.6	0.743
Gender (male, n [%])	32 (86.5)	51 (87.9)	83 (87.4)	0.836
Cigarette smoking, n (%)	23 (62.2)	39 (67.2)	62 (65.3)	0.612
Alcohol consumption, n (%)	12 (32.4)	27 (46.6)	39 (41.1)	0.175
Hypertension, n (%)	28 (75.7)	30 (51.7)	58 (61.1)	0.022
Diabetes mellitus, n (%)	11 (29.7)	17 (29.4)	28 (29.5)	0.965
Coronary heart disease, n (%)	2 (5.4)	7 (12.1)	9 (9.5)	0.292
Hyperlipidemia, n (%)	19 (45.9)	32 (55.2)	51 (53.7)	0.335
Blood glucose (mmol/L)	6.7 ± 2.5	6.0 ± 11.2	6.3 ± 2.5	0.214
Total cholesterol (mmol/L)	4.7 ± 3.4	4.8 ± 1.0	4.8 ± 1.1	0.758
Uric acid (umol/L)	345.4 ± 120.4	349.2 ± 73.3	346.5 ± 94.3	0.734
Homocysteine (umol/L)	19.5 ± 13.7	18.4 ± 15.0	18.8 ± 14.2	0.714
Folic acid (ng/mL)	6.4 ± 2.4	7.0 ± 4.2	6.7 ± 3.6	0.418
SBP (mmHg)	146 ± 19	140 ± 22	142 ± 21	0.131
DBP (mmHg)	81 ± 13	80 ± 15	80 ± 14	0.822
Heart rate (bpm)	69.3 ± 10.3	71.0 ± 9.5	70.4 ± 9.8	0.400
Average Phase (degree)	40.6 ± 21.9	33.0 ± 15.4	36.0 ± 18.5	0.028
Average Gain (%/%)	0.7 ± 0.3	0.8 ± 0.3	0.8 ± 0.3	0.141
Total CSVD burden				0.106
0	4 (10.8)	3 (5.1)	7 (7.4)	-
1	9 (24.4)	14 (24.1)	23 (24.2)	-
2	15 (40.5)	15 (25.9)	30 (31.6)	-
3	5 (13.5)	15 (25.9)	20 (21.0)	-
4	4 (10.8)	11 (19.0)	15 (15.8)	-

Notes: Data are expressed as mean ± standard deviation/median and interquartile range or n (%). Abbreviations: DMV, deep medullary vein; NIHSS, national institutes of health stroke scale; SBP, systolic blood pressure; DBP, diastolic blood pressure; CSVD, cerebral small vessel disease.

### 3.2 Association between DMV scores and total CSVD burden

In the univariate linear regression analyses, the total DMV scores were correlated significantly with the total CSVD burden (β = 0.23, *p* = 0.025). After adjusting for possible confounding factors, multivariate analysis (Table 2) showed that this positive correlation remained significant.

**TABLE 2:** Multivariate analysis of DMV scores and total CSVD burden/phase.

	Total DMV scores and total CSVD burden		DMV (left) and phase (left)		DMV (right) and phase (right)		Total DMV scores and average phase	
	β (95CI)	P	β (95CI)	P	β (95CI)	P	β (95CI)	P
Model 1	0.23 (0.01, 0.11)	0.025	−1.61 (−3.20, −0.02)	0.047	−1.62 (-3.19, −0.02)	0.046	−0.81 (−1.60, −0.01)	0.046
Model 2	0.05 (0.01, 0.10)	0.043	−1.72 (−3.38, −0.05)	0.044	−1.75 (−3.36, −0.13)	0.034	−0.88 (−1.70, −0.06)	0.035
Model 3	0.06 (0.01, 0.12)	0.022	−1.84 (−3.61, −0.06)	0.042	−1.78 (−3.49, −0.06)	0.043	−0.92 (−1.79, −0.04)	0.040
Model 4	0.06 (0.01, 0.12)	0.041	−2.20 (−4.06, −0.30)	0.023	−2.25 (−4.03, −0.48)	0.014	−1.13 (−2.05, −0.22)	0.016
Model 5	0.08 (0.01, 0.14)	0.019	−2.30 (−4.57, −0.75)	0.023	−2.21 (−4.06, −0.37)	0.019	−1.14 (−2.09, −0.18)	0.020

Model 1: univariate; Model 2: adjusted for age, gender, cigarette smoking and alcohol consumption; Model 3: adjusted for the variables in model 2, as well as hypertension, diabetes mellitus, coronary heart disease and hyperlipidemia; Model 4: adjusted for the variables in model 3, as well as systolic blood pressure, diastolic blood pressure and heart rate; Model 5: adjusted for the variables in model 4, as well as blood glucose, total cholesterol, uric acid, homocysteine and folic acid.

### 3.3 Association between DMV scores and dCA

In the univariate linear regression analyses, DMV scores in the left hemisphere were correlated significantly with the phase measured in the left hemisphere (β= −0.20, *p* = 0.047). There was a similar significant linear correlation between DMV scores and the phase in the right hemisphere (β= −1.62, *p* = 0.046). The total DMV scores also correlated with the average bilateral phase (β= −0.81, *p* = 0.046). In multivariate analysis, all models showed a negative correlation between DMV scores and the phase (Table 2) after adjusting for possible confounding factors. No significant association was found for the gain parameter; therefore, the results are not shown.

### 3.4 Effect of dCA on the association between DMV scores and total CSVD burden

**FIGURE 4:**
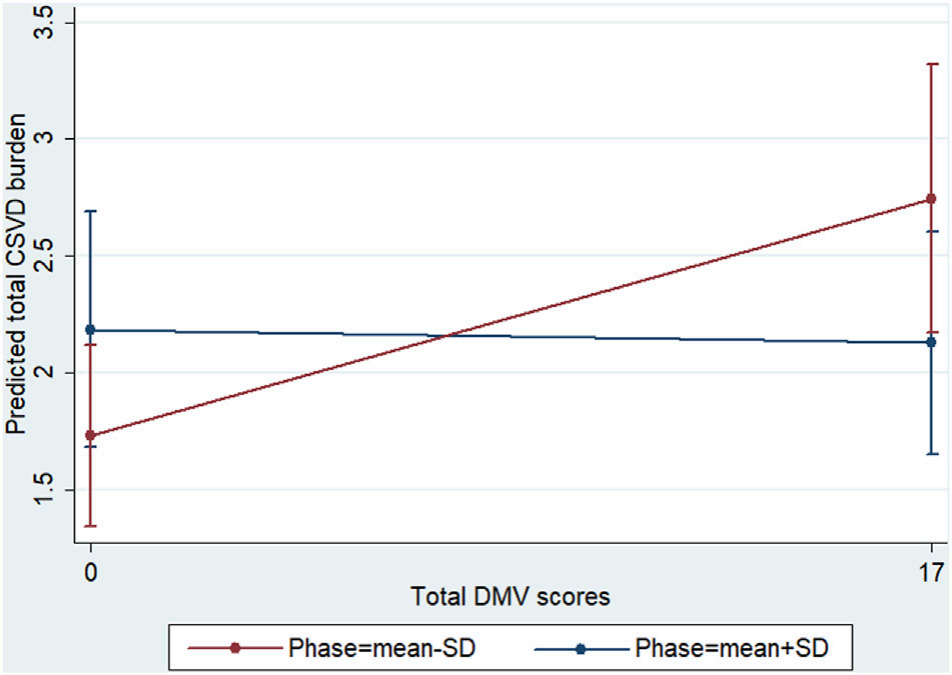
Interaction diagram. Predictive marginal means of the total CSVD burden with total DMV scores and phase stratified at the lower (mean −SD) and higher (mean +SD) phases. The confidence intervals of the margins of the total CSVD burden overlapped when the higher phase was compared across the total DMV scores. When the lower phase was compared across the total DMV scores, the predicted confidence interval was significant (total CSVD score margins: 1.73 [95% CI, 1.34–2.12] vs. 2.75 [95% CI, 2.17–3.32], *p* < 0.001).

To further investigate whether dCA plays a role in the association between DMVs and CSVD, an interaction analysis was conducted. The interaction model demonstrated a significant positive interaction effect of dCA on the aforementioned association (β = 0.003 for the interaction term; *p* = 0.02). As shown in Figure 4, the predicted confidence interval of margins of the total CSVD score was not statistically significant when the higher phase (mean +SD) was compared across the total DMV scores. In contrast, the predicted confidence interval was significant when the lower phase (mean −SD) was compared across the total DMV scores (total CSVD score margins: 1.73 [95% CI: 1.34–2.12] vs. 2.75 [95% CI: 2.17–3.32], *p* < 0.001). These results suggest that lower phase values (which represent dCA impairment) enhanced the association between DMV scores and total CSVD burden.

## 4 Discussion

In this prospective observational study, we found an association between DMV scores and total CSVD burden, indicating that higher DMV scores are associated with a more severe CSVD burden. More importantly, to the best of our knowledge, this is the first study to show that DMV scores have a strong negative correlation with dCA function, independent of demographic and vascular risk factors. Furthermore, dCA impairment was found to have a positive interaction effect on the association between DMV scores and the total CSVD burden.

Previously, CSVD was thought to be caused by perforating artery occlusion, chronic hypoperfusion, or arterial leakage. However, some venous pathologies behind CSVD have been found in recent years. Moody et al. first identified and defined periventricular venous collagenosis, a stenosis and occlusion of veins, in 1995 (Moody et al., 1995). In 2014, François et al. observed a reduction in the number of visible veins in patients with cerebral autosomal-dominant arteriopathy with subcortical infarcts and leukoencephalopathy (De Guio et al., 2014). In 2017, Keith et al. (2017) found that periventricular venous collagenosis was encountered frequently in histologic specimens of CSVD patients, and that venous collagenosis was strongly associated with WMH. In recent years, research has shown that DMV changes are strongly associated with CSVD image features. However, these results are not completely consistent. Xu et al. (2020) found that DMV scores were correlated closely with WMH, lacunae, an enlarged periventricular space, and total CSVD burden in CSVD patients; however, Chen et al. (2020) found that DMV scores correlated with WMH, lacunae, and brain atrophy but did not correlate with total CSVD burden in patients with atherosclerotic CSVD. Ao et al. (2021) did not find any correlation between DMV scores and CSVD images, except for brain atrophy, in a large community-based cohort. These inconsistencies might be due to the different study designs and populations. In the present study, we chose the total CSVD burden to represent the severity of CSVD and observed a close relationship between the DMV scores and total CSVD burden. As the DMV scores increased, the total CSVD burden also increased. This result is consistent with the findings of Xu et al. (2020).

We found an inverse correlation between DMV scores and dCA function, after adjusting for possible confounding factors. Because no relevant studies have been conducted, the mechanisms of this correlation remain largely unknown. Combined with anatomical physiology, we hypothesize three possible explanations. 1) Stenosis or occlusion of the venous lumen: deep medullary venous collagenosis or insufficient venous clearance can cause venous lumen stenosis or occlusion. Multiple arterioles have been found to rely on a single venule for drainage in humans (Hartmann et al., 2018); venous stenosis may affect arteriole drainage and thus impair dCA. 2) Increased venule tortuosity: venule tortuosity has been observed both in mouse models and *in vivo*. In 2015, tortuosity alterations in penetrating venules were found in a mouse model of Alzheimer’s disease (Lai et al., 2015). In 2017, Bouvy et al. observed that the tortuosity of DMVs was more severe in patients with mild cognitive impairment and early Alzheimer’s disease than in age-matched control individuals (Bouvy et al., 2017). Vessel structural changes, such as increased tortuosity, can reduce vessel reactivity (Farooq and Lee., 2021), change vascular shear stress, and disturb hemodynamics, which can ultimately impair dCA. 3) Disruption of neurovascular units: the achievement of dCA mainly depends on neurovascular units, which consist of arterioles, venules, endothelial cells, and other components (Wang et al., 2015). Previous studies found that inflammation is involved in the development of DMV disruption (Zhang et al., 2022b); the inflammatory response causes vascular endothelial damage (Shaaban et al., 2019) and results in dCA impairment.

**FIGURE 5:**
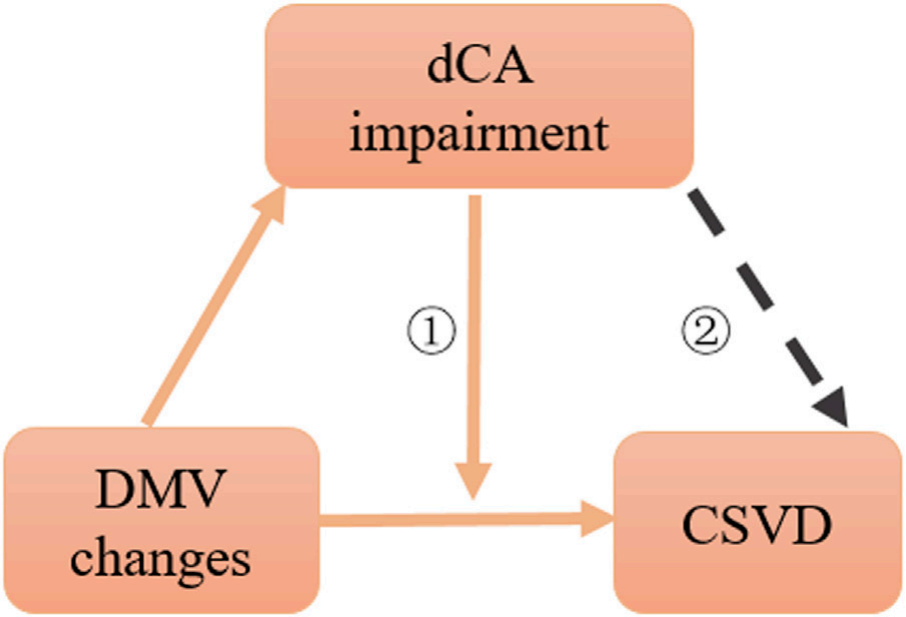
Schematic diagram of association among dCA, DMV changes, and CSVD. ①: interaction effect, ②: citation from CNS Neurosci Ther. 2022; 28:298–306.

The present study showed that DMV changes were associated with dCA impairment, and our previous study confirmed that dCA impairment is correlated with the severity of CSVD; however, weather dCA was a promotor between DMV and CSVD remain unclear. Thus, we further explored the effect of dCA impairment on the association between DMV scores and the total CSVD burden. We found that a lower dCA had an interactive effect on this aforementioned association, suggesting that the effect proportion of DMV changes on the total CSVD burden relies on the degree of dCA impairment. When DMV changes, not only CSVD but also dCA function can be affected. Therefore, part of the reasons for the interactive effect is that DMV changes affect CSVD is partly realized by affecting dCA function. DMV changes and dCA impairment have synergistic effect on CSVD (Figure 5).

Our study has some limitations. First, the DMV scores were assessed visually, which might have affected repeatability. Second, this was a cross-sectional study, and we could not exactly prove the causal relationship among DMV changes, dCA impairment, and CSVD burden. Third, this was a single-center study with a relatively small sample size; therefore, there may have been some selection bias. Our study also lacked follow-up data; therefore, we could not assess the longitudinal progression of CSVD. Further research in this area will be conducted in the future.

In summary, DMV changes were independently correlated with CSVD burden and dCA impairment. Furthermore, impaired dCA function had a significant positive interaction effect on the association between DMV changes and the total CSVD burden. Our study provides new insights into the correlation among changes in DMV, total CSVD burden, and dCA function. These results can help improve the understanding of the complex pathogenesis and progression of CSVD, thereby facilitating early intervention and treatment development. However, further studies are required in the future.

## Data Availability

The raw data supporting the conclusion of this article will be made available by the authors, without undue reservation.
